# Grasp and remember: the impact of human and robotic actions on object preference and memory

**DOI:** 10.1038/s41598-024-70692-0

**Published:** 2024-08-27

**Authors:** Alex Kafkas, Megan Rowland, Paolo Gallina, Luca F. Ticini

**Affiliations:** 1 Division of Psychology, Communication and Human Neuroscience, School of Health Sciences, University of Manchester, Manchester, UK; 2 Department of Civil Engineering and Architecture, University of Trieste, Trieste, Italy; 3 Department of Psychology, Webster Vienna Private University, Vienna, Austria

**Keywords:** Action observation, Goal contagion, Mimetic desire, Preference, Recognition memory, Recollection, Human behaviour, Long-term memory, Motor control, Sensorimotor processing

## Abstract

Goal contagion, the tendency to adopt others' goals, significantly impacts cognitive processes, which gains particular importance in the emerging field of human–robot interactions. The present study explored how observing human versus robotic actions affects preference and memory. Series of objects undergoing either human or robotic grasping actions together with static (no action) objects were presented, while participants indicated their preference for each object. After a short delay, their memory for grasped, static and new (unstudied) stimuli was tested. Human actions enhanced preference and subsequent recollection of objects, more than robotic actions. In the context of human action, static objects were also perceived as more familiar at recognition. The goal contagion's influence on memory was found to be independent from its impact on preference. These findings highlight the critical role of human interaction in eliciting the impact of goal contagion on cognitive evaluations, memory engagement and the creation of detailed associative memories.

## Introduction

In our modern society, marked by pervasive advertising, extensive consumerism, and impulsive buying behaviours, the examination of goal contagion^1,2^ assumes significant pertinence and timeliness. Goal contagion broadly refers to the intrinsic inclination of individuals to unknowingly adopt the goals of others as their own, thereby allowing their behaviour to be influenced by the actions they observe^3,4^. This phenomenon has far-reaching implications as it may shape our evaluation of situations and decision-making processes, and even memory. A more targeted aspect of goal contagion relates directly to preference formation and is known as mimetic desire^5^. Mimetic desire specifically refers to the propensity to prefer and desire what other individuals pursue, reflecting a focused influence of goal contagion on preference.

Insofar, research has yet to systematically explore the impact of goal contagion on memory. Goal contagion may impact memory by changing the recall and recognition of objects associated with observed goals, whereas mimetic desire may primarily influence the subjective valuation and preference for those objects. Therefore, the present study aims to investigate the effect of actions towards objects on their preference (mimetic desires) and subsequent memorability. Additionally, we explored whether the type of hand performing the action (human versus robotic) has differential contribution to the preference and memory effects.

### Goal contagion and mimetic desire

The neural bases of goal contagion are found within the parietal, premotor, and middle temporal areas of the Action Observation Network (AON), activated both when performing an action and when observing someone else performing that action^6,7^. This function is the basis for what is often referred to as the Mirror Neuron System^8–10^, since these neurons 'mirror' the behaviour of others, as though the observers were themselves acting. Action mirroring is thought to underlie the comprehension and interpretation of others' actions and intentions, thereby serving as a cornerstone of social cognition^10^.

Goal contagion underpins mimetic desire, the propensity to prefer and desire what other individuals pursue^11^. A compelling illustration of this tendency is witnessed in virtual marketplaces, wherein consumers fervently compete to acquire the same fashionable product, despite the availability of a diverse range of equally enticing alternatives. Research employing brain imaging and neuromodulation techniques^12,13^ suggests a pivotal role of the AON in forming mimetic desires when others’ actions are observed, notably through the regulation of the activity of the Brain Valuation System (BVS). The BVS, which includes the ventral striatum and the ventromedial prefrontal cortex^14,15^, encodes the perceived worth of objects^5,16–18^. As such, the interplay between the AON and BVS offers insight into how goal contagion influences short-term preferences in mimetic desire.

### Memory processes and goal contagion

Goal contagion may also affect memory functions, but this link has not been investigated systematically. Recognition memory, the ability to determine whether a stimulus has been encountered before or not, relies on two memory types that vary in the extent to which they involve accessing information from memory^19–21^. Familiarity refers to the feeling that a stimulus has been previously encountered without recalling specific contextual details of the encounter. On the other hand, recollection involves retrieving additional information associated with the cueing stimulus. These memory types are influenced to varying degrees by the availability of different environmental cues and are affected by different manipulations^20,22,23^. Notably, observing goal-oriented actions of others serves as one such influential cue. As demonstrated by Lacoste-Badie and Droulers^24^, the act of witnessing an object as the intended goal of another's actions can enhance recall and recognition of that object. This suggests the presence of an implicit link between memory processes and the phenomenon of goal contagion. However, a comprehensive understanding of how goal contagion and mimetic desires interweave with the intricate processes of memory formation, retention, and retrieval, remains to be explored. Firstly, it remains unclear whether observing a goal-directed action affects long-term memory formation and leads to different memory experiences. Additionally, previous studies investigating the link between observing goal-directed actions and enhanced memory for objects have often focused on a limited number of stimuli e.g.,^24^. Therefore, the present study aimed to expand on this research by examining recognition memory of various household objects, including tools and non-tools.

### Human and robotic agents

A novel aspect of contemporary research is the examination of the involvement of the AON in actions performed by robotic systems^25^. As society continues to incorporate robots at an unprecedented scale, gaining an understanding of how humans perceive and react to these non-human actors' actions becomes increasingly critical. Pioneering studies, as reviewed by Press^26^ indicate that the AON's response is not exclusive to human object manipulation, but also extends to similar actions performed by industrial robots, see also^27^. This finding was further corroborated by an EEG study which revealed mu-suppression in the AON regions in response to both robots and humans^28^. This hints at the possibility that goal contagion for robots might be processed similarly to that for humans, warranting further research into the role of mimetic desires and memory functions in this interaction.

### The present study

Expanding upon these emerging insights, our current investigation aimed to bring together these varied areas of research. We focused on examining the impact of goal contagion and mimetic desires on memory formation and retrieval, and notably, we contrasted these processes in human–human and human–robot interactions. Furthermore, the study incorporated item-by-item familiarity and recollection measures at recognition to explore differences in memory experiences associated with objects subjected to actions by different agents. Our research sought to unravel the complex interplay of these components, thereby bridging a gap in our current understanding of these phenomena. In doing so, we hope to illuminate aspects of social cognition, an area of study gaining increasing relevance in our rapidly evolving, technology-driven society.

Building upon previous research, our hypothesis was that participants would exhibit increased preference and memory performance for objects when they were encoded with a grasping action compared to when the objects were presented statically. Furthermore, the study sought to explore whether the type of hand (human or robotic) and the type of action (grasping or static) performed towards an object during encoding influenced the preference and memorability of the object. We hypothesized that differences would exist in object preference and memory performance between participants who viewed a human hand grasping an object during encoding and those who observed a robotic hand performing the same action. Additionally, we anticipated that object preference and memory performance would be influenced by both the type of agent (human or robotic) and the type of action (grasping or static) involved at encoding. Moreover, using mediation analysis, we investigated whether any differences in memory performance resulting from action observation during encoding were independent or driven by changes in preference reported at encoding. This allowed us to explore the potential mechanisms through which action observation affects both preference and memory.

## Methods

### Ethical approval and informed consent

Ethical approval for the study was secured by the University of Manchester Proportionate Ethics Committee. The committee adheres to international standards (such as the Helsinki Declaration), national standards (such as those set by the Health Research Authority) and standards from professional bodies (such as the British Psychological Society), related to collecting data from human participants. Participants in the experiment read a participant information sheet (PIS) and each provided informed consent online prior to starting the experiment.

### Participants

Participants in the study were psychology students at the University of Manchester, who completed the experiment online (gorilla.sc) and received course credits upon completion of the experiment. They were all native English speakers, with normal or corrected to normal vision, with no neurological or psychiatric conditions, and who were not taking any psychotropic medication. Sixty participants were originally recruited to take part in the study, whereby 30 were allocated to the robotic-hand condition, while 30 were allocated to the human-hand condition. As 15 participants had to be excluded from parts of the study due to an error with data recorded at the recognition memory task, an additional 15 participants were recruited (total n = 75). The error in the original 15 participants was due to the use of incompatible devices (tablets and mobile phones), which prevented recording their responses in the recognition memory task. From this sample, a total of 74 participants were included in the encoding/preference rating task with 38 in the robotic-hand condition (mean age = 19.18, SD = 1.01 years; 33 female) and 36 in the human-hand condition (mean age = 19.14, SD = 1.10 years; 29 female). In the recognition memory task, 58 participants were included in the analyses with 28 in the robotic-hand condition (M age = 19, SD = 1.01 years), and 30 participants in the human-hand condition (M age = 19, SD = 1.12 years).

### Materials and stimuli

**Fig. 1:**
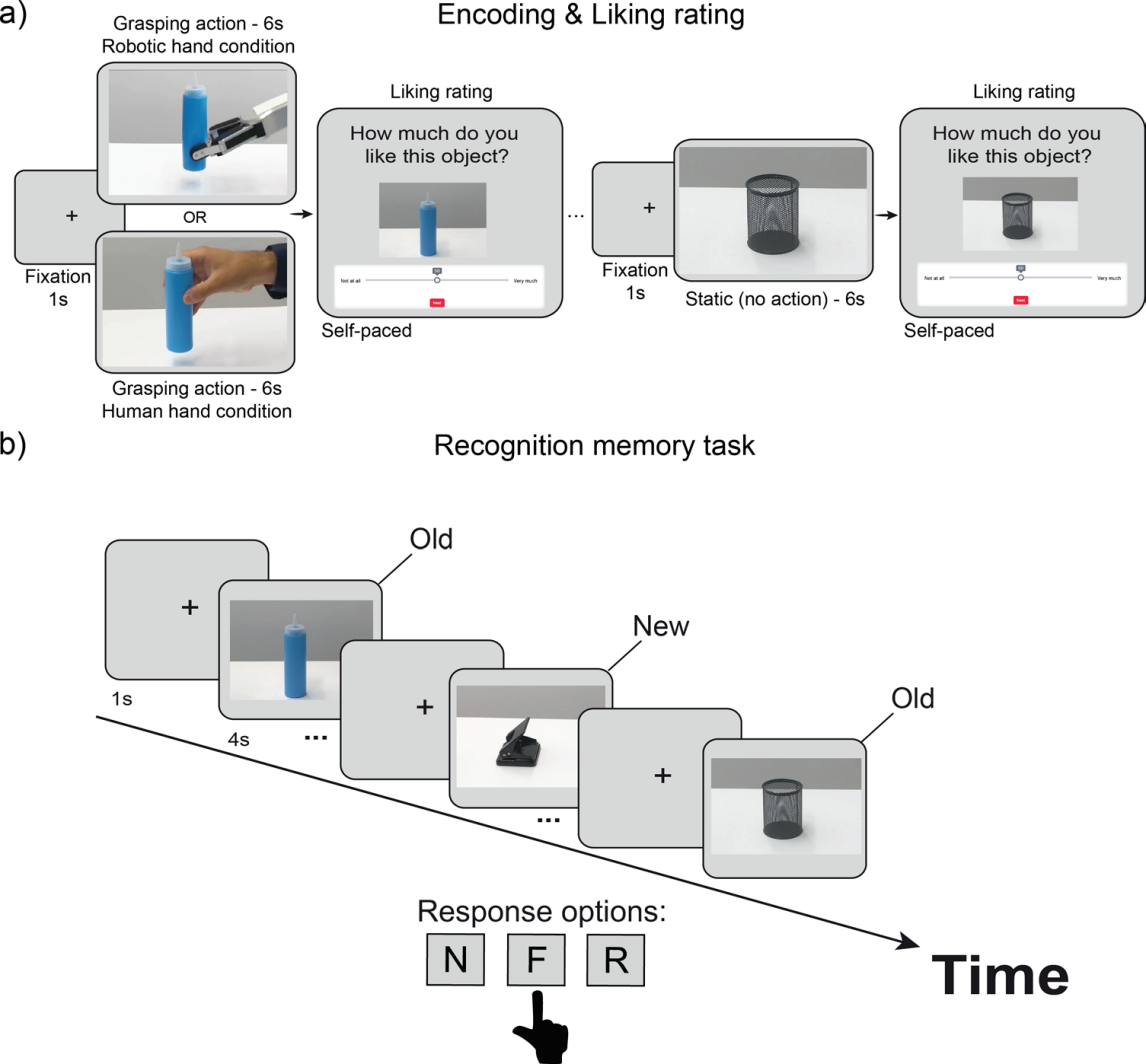
Design of the experiment. (**a**) At encoding participants were exposed to either a robotic-hand or a human-hand condition with grasping actions towards the objects. Static objects (without any action) were also presented in both conditions. Participants indicated how much they liked each object (from ‘not at all’ to ‘very much’) using a slider. (**b**) At recognition, participants provided new (N), familiar (F) or recollected (R) responses to previously studied and unstudied items.

In the encoding/preference task, 40 objects were selected from a pool of 60 for each participant session. In the recognition memory task, participants viewed the 40 studied objects presented at encoding (old) and an additional 20 new (previously unstudied) objects. The allocation of objects at encoding was randomised for each participant, with the only restriction that 20 action and 20 non-action (static) objects were shown. Objects, which were developed for this study, included neutral/everyday items without explicit emotional content. For example, food (e.g., apple) or household items (e.g., a jar). Video stimuli with 60 objects were created in three versions (Fig. 1). The human grasping version presented each object on the table, while a hand was reaching to it and grasped it from either the top or the side. The robotic grasping version included the same actions but performed by a robotic hand. In both cases the rest of the agent (body/face) were not visible to eliminate the impact of eye gaze on the participants’ judgments^29^. In each video, the object was displayed on a table for the first three seconds, with no visible agent. Subsequently, a hand (human or robotic) appeared from the right side of the frame and performed the action over the next three seconds. This setup was consistent across both the human and robotic-hand conditions. Finally, the static version was a video recording of each object without any action, no visible human or robotic hand and no movement to the object. The object was presented lying on the table for the duration of the clip. All videos had the same duration of six seconds each. After the encoding/preference block, participants completed a filler task with 35 arithmetic calculations, which lasted for approximately five minutes. The Gorilla platform (https://gorilla.sc) was used to collect data online.

### Design and procedure

A 2 × 2 mixed design was used. The two independent variables were hand condition (robotic or human; between-subjects) and action (grasped or static; within subjects). The two dependent variables we measured included i) object preference and ii) memory performance separately for each memory type (familiarity and recollection). Participants completed the task online, but they were instructed at the beginning of the study to complete the experiment in one sitting and in a quiet space free from any distraction. At the start of the experiment, each participant read the participant information sheet, consented to take part, and provided demographic data (age and gender). They were randomly allocated to either the human-hand condition or the robotic-hand condition and each participant completed three tasks/blocks: an encoding/preference task, a filler and a recognition memory test.

Encoding/preference task: In this task, participants saw a series of videos showing an object on a white table to be grasped by an agent (human or robotic hand—in separate conditions) or to lie static on the table with no action performed on them. Participants were instructed to observe the objects carefully and to provide a preference rating after the end of the video, without any reference to the subsequent memory task. After each video, participants were presented with an image of the studied object (see Fig. 1) and were asked to use a preference scale to indicate how much they liked the object by moving their cursor along a 0–100 preference rating scale situated below each object (0 = ‘not at all’; 100 = ‘very much’). Previous studies have shown that the precise formulation of the question (e.g., “How much do you like to use the object?” or “How much would you like to acquire the object?”) is not of importance^12,13^. Therefore, for each object the question ‘how much do you like this object’ was used. The preference rating was self-paced and after indicating their preference, the next trial appeared on the screen. A total of 40 objects were presented that way, with 20 objects in the grasping and 20 in the static condition and each video lasted for six seconds. Each trial commenced with a one-second fixation cross, succeeded by a six-second video showing either a grasped or static object and ended with the participant's self-paced preference rating while the image of the object was on the screen.

Filler task: After the encoding task, each participant completed 35 arithmetic calculations including additions, subtractions and multiplications. They were instructed to select the ‘correct’ or ‘incorrect’ buttons with their mouse depending on whether they believed the provided result was accurate or not. They were asked to prioritise accuracy over speed and instant feedback was provided after indicating their response in each trial. Each trial consisted of a fixation cross (one second), followed by the self-paced arithmetic problem.

Recognition memory task: Before starting this block, participants were instructed that their memory for the objects presented in the first block of the study would be tested and that a combination of old (studied) and new stimuli would be presented. They were instructed to report old stimuli by pressing either the ‘familiar’ or ‘recollected’ button appearing on the screen or the ‘new’ button for unstudied objects. They were trained to discriminate instances of familiarity and recollection and their understanding of these memory experiences was tested by providing hypothetical scenarios and asking them to classify the experience as indicating either familiarity or recollection. Corrective feedback was given, in case of inaccurate responses, while participants had the option to read the initial instructions again. The instructions explained that a familiarity response indicates a feeling of memory (familiarity) for the object itself, without being able to retrieve anything additional from the previous encounter. This memory type indicates a sense of recognition from a previous encounter without recalling any further specific details. On the other hand, they were told that recollection indicates that an item is recognised based on recalling associative details from the time of first encounter, which could include their thoughts while an object was on the screen or any other associative detail from encoding^20–22,30^. Each trial started with a fixation cross (one second) followed by an object (four seconds), during which participants could select a ‘new’, ‘familiar’ or ‘recollected’ button presented at the bottom of the screen. In total, 60 trials were presented in this block with 40 studied stimuli (from encoding) and 20 new foils.

### Data analyses

JASP statistical package (JASP Version 0.17.2) was used for all statistical analyses. Familiarity memory performance was calculated using the sensitivity or discriminability index d’ calculated as the standardised hit rate minus the standardised false alarm rate (d′ = *z*(HIT) − *z*(FA))^31^. Recollection performance was calculated as proportion of hits—proportion of FAs, as this is usually the preferred measure for recollection, which is considered to be a threshold process as opposed to a strength-based signal detection process (as assumed when calculating d′)^21^. Nevertheless, the findings were very similar when recollection d’ was used in the analyses. As the relationship between the two memory kinds is still debated (independence or exclusivity), familiarity rates (and d’) were calculated both for exclusivity (i.e., all F responses reflect familiarity only and all R responses recollection only) and for independence using the following formula: F_independence_ = F_proportion_/(1 − R_proportion_)^32^. Preference and memory performance (separately for familiarity d’ independence and recollection) were analysed using mixed two-way ANOVAs with hand type (robotic and human) as a between subjects’ factor and action type (grasped and static) as a within subjects’ factor. Significant interactions were further explored using Bonferroni-corrected post-hoc comparisons. As the familiarity d’ measure in the human-hand condition was not normally distributed, the effects of action type (static versus grasped) across the two hand conditions (human versus robotic) were explored using non-parametric statistical tests (the Wilcoxon signed-rank test and the Mann–Whitney *U* test). A significance level of α = 0.05 was adopted for all tests, while partial eta-squared (η_p_^2^) or the rank biserial correlation (r_rb_; for non-parametric tests) are reported as measures of effect size. Mediation analyses^33^ were conducted to explore whether the effect of action at encoding on subsequent memory performance was mediated by changes in preference. Separate mediation analyses were conducted for human and robotic conditions and across the different outcome memory measures (i.e., familiarity d’, familiarity d’_Independence_, recollection performance). To estimate the mediation parameters, the maximum likelihood estimation (MLE) method was used within the framework of moderation analysis. The MLE provided the point estimates for the direct and indirect effects in the model. To assess the precision of these estimates, bias-corrected percentile bootstrap confidence intervals (with 1,000 bootstrap samples) were employed, and these are reported when assessing the indirect effects^34^. A 95% confidence level was used for the bootstrap confidence intervals, indicating that there is a 95% probability that the intervals contain the true parameter values. For the direct effects, both the percentile confidence intervals and the *p*-values, derived using Delta method standard errors, are reported.

## Results

### Preference

**Fig. 2:**
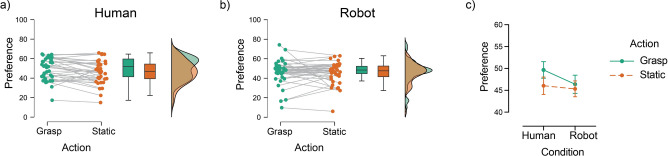
The effect of action on preference in the human-hand and robotic-hand conditions. (**a**, **b**) Raincloud plots showing individual preference responses, box plots (with median and interquartile range) and violin plots (distribution of preference responses) for grasped and static objects in the two hand conditions. (**c**) Descriptive/summary plot of the overall effect of action on preference for human-hand and robotic-hand conditions. Error bars show ± 1 standard error of the mean.

Preference ratings across participants and conditions are presented in Fig. 2a,b (see Fig. 2c for overall effects). The two-way ANOVA on preference ratings indicated a significant main effect of action type (*F*_1,72_ = 4.09, *p* = 0.047, η_p_^2^ = 0.054) showing increased preference for grasped objects (M = 48, SE = 1.38) relative to static ones (M = 45.66, SE = 1.38; Fig. 2c). However, the hand type had no significant effect on preference ratings (main effect of hand type* F*_1,72_ < 1). Visual inspection of the plots in Fig. 2, shows that the effect of action appears to be stronger in the human-hand condition, however, this did not reach statistical significance (action by hand type interaction: *F*_1,72_ = 1.24, *p* = 0.27, η_p_^2^ = 0.017). Overall, these effects indicate that grasping actions lead to increased desirability of objects irrespective of the human/robotic characteristics of the agent.

### Memory performance

**Fig. 3:**
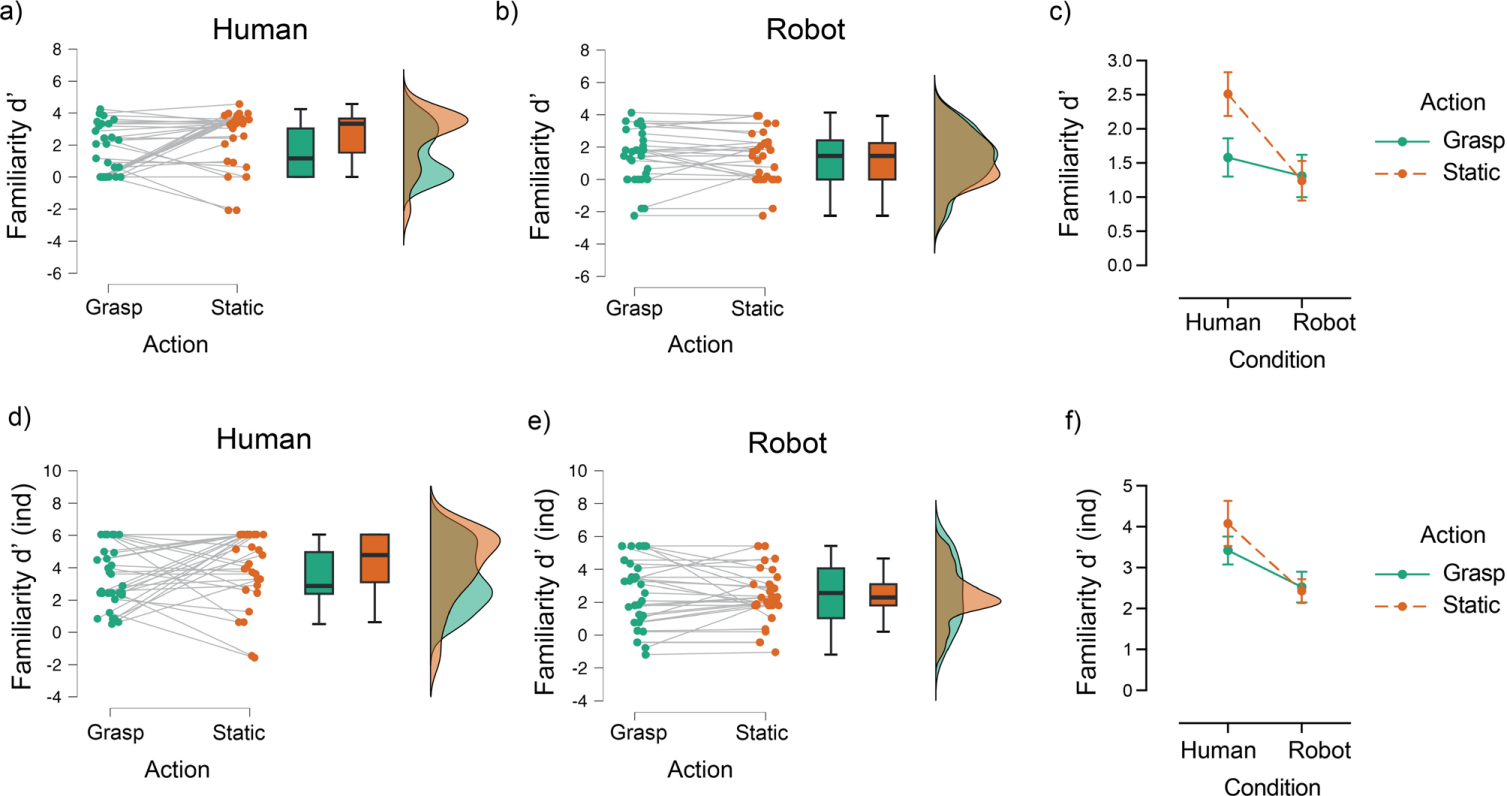
The effect of action on familiarity performance (d′) for grasped and static objects in the human-hand and the robotic-hand conditions. (**a**, **b**) Raincloud plots showing individual familiarity scores, box plots (with median and interquartile range) and violin plots (distribution of familiarity scores) for grasped and static objects in the two hand conditions (**c**) Descriptive plot of the overall effect of action on familiarity d’ for human-hand and robotic-hand conditions. Error bars show ± 1 standard error of the mean. The same effects on familiarity d’ calculated under the criterion of independence are presented in panels (**d**–**f**).

**Fig. 4:**
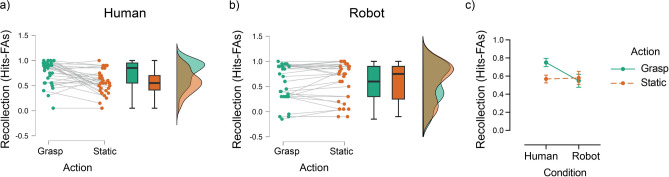
The effect of action on recollection performance (Hits—FAs) for grasped and static objects in the human-hand and the robotic-hand conditions. (**a**, **b**) Raincloud plots showing individual recollections scores, box plots (with median and interquartile range) and violin plots (distribution of recollection scores) for grasped and static objects in the two hand conditions (**c**) Descriptive plot of the overall effect of action on recollection performance for human-hand and robotic-hand conditions. Error bars show ± 1 standard error of the mean.

Familiarity d’, d’_Independence_ and recollection performance (hits—FAs) across participants and conditions are presented in Figs. 3 and 4. The analyses on familiarity d’ showed higher familiarity performance for static (M = 2.52, SD = 1.80) than grasped objects (M = 1.58, SD = 1.55) in the human-hand condition (Wilcoxon *W* = 265.5, *p* = 0.028, r_rb_ = 0.50), but no significant differences between static (M = 1.24, SD = 1.58) and grasped objects (M = 1.31, SD = 1.68) in the robotic-hand condition (Wilcoxon *W* = 82.5, *p* = 0.63). Further comparing familiarity d’ for static objects between the two hand conditions (human versus robotic) revealed significantly higher familiarity performance for static items in the human hand (M = 2.52, SD = 1.80) than the robotic-hand condition (M = 1.24, SD = 1.58; *U* = 243, *p* = 0.002, r_rb_ = -0.46). Conversely, the same comparison for grasped objects did not reveal any significant differences between the two hand conditions (human: M = 1.58, SD = 1.55; robotic: M = 1.31, SD = 1.68; *U* = 423, *p* = 0.69). Therefore, as shown in Fig. 3c, static objects in the human-hand condition were characterised by higher familiarity than grasped objects in the same condition, while the robotic condition had no differential effect on familiarity for grasped versus static objects . Nevertheless, the analysis with familiarity d’ _Independence_ (calculated using the criterion of independence) only showed a significant main effect of hand type (*F*_1,58_ = 9.54, *p* = 0.003, η_p_^2^ = 0.141), with higher overall reported familiarity in the human (M = 3.75, SE = 0.29) than in the robotic condition (M = 2.48, SE = 0.30; Fig. 3d–f). All the other effects were not statistically significant (main effect of action type: *F*_1,58_ = 0.94, *p* = 0.34; action type by hand type interaction: *F*_1,58_ = 1.68, *p* = 0.20).

The analysis on recollection performance (hits—FAs), revealed a significant main effect of action type (*F*_1,55_ = 5.88, *p* = 0.019, η_p_^2^ = 0.097), with greater recollection performance for grasped (M = 0.65, SE = 0.04) than static objects (M = 0.58, SE = 0.04), while the main effect of hand condition was not significant (*F*_1,55_ = 1.56, *p* = 0.22, η_p_^2^ = 0.028). Importantly, the action type by hand condition interaction was significant (*F*_1,55_ = 11.84, *p* = 0.001, η_p_^2^ = 0.18), stemming from the increased recollection accompanying grasped objects relative to static ones in the human-hand condition (*p*_*bonf*_ < 0.001), but no difference between the two actions in the robotic-hand conditions (*p*_*bonf*_ = 1.00*; *Fig. 4c). Therefore, grasping actions by a human hand increased subsequent recollection for these objects, while at the same time boosted familiarity for the static objects in the same condition (when exclusivity is assumed). These effects were selective to (or more prominent in) the human-hand condition.

### Mediation analysis

**Fig. 5:**
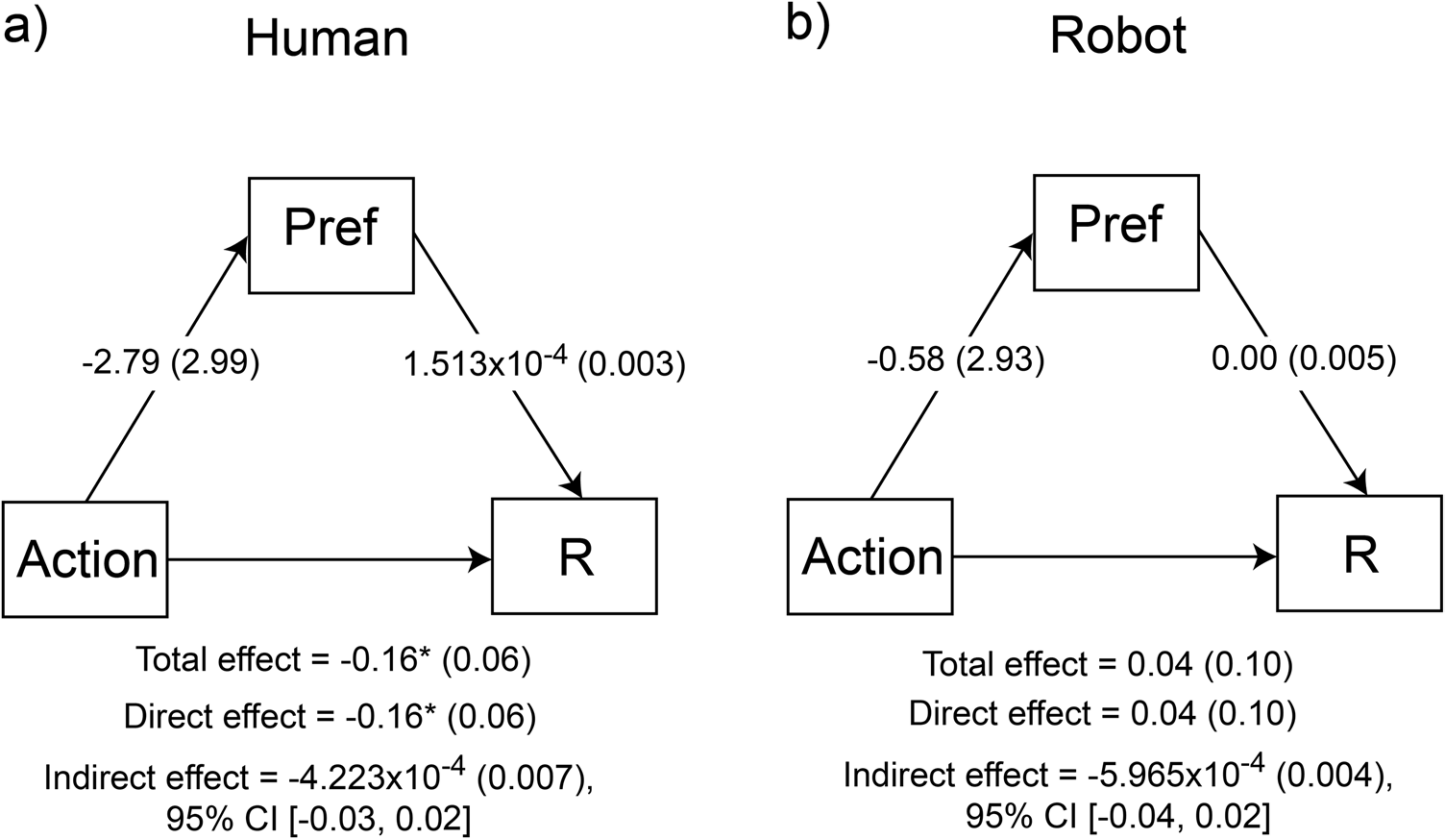
Mediation analysis of the effect of action on recollection performance (R), as a direct effect or as an indirect effect mediated by preference in the two conditions; (**a**) human hand; (**b**) robotic hand. Coefficients represent regression coefficients with standard errors in parenthesis. Pref = preference, *p < 0.05.

**Fig. 6:**
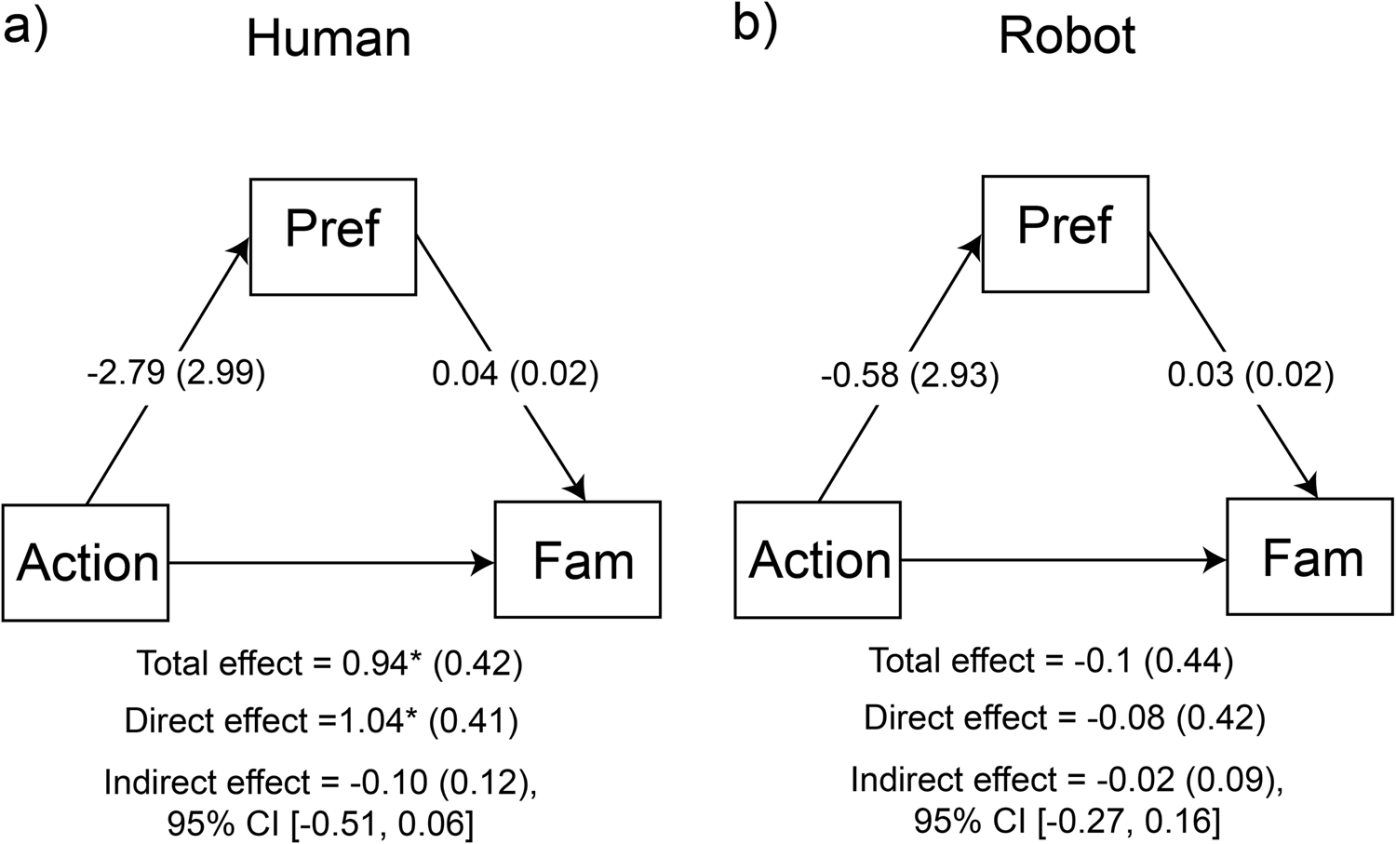
Mediation analysis of the effect of action on familiarity performance (Fam), as a direct effect or as indirect effect mediated by preference in the two conditions; (**a**) human hand; (**b**) robotic hand. Coefficients represent regression coefficients with standard errors in parenthesis. Pref = preference, *p < 0.05.

In the analyses presented so far, grasping actions at encoding appeared to modulate the desirability of an object (as indicated by preference), while at the same time increased subsequent recollection performance. The next question we explored was the extent to which the effect of action on subsequent memory was mediated by the increased preference. In other words, we questioned whether the increased preference associated with grasped objects resulted in stronger encoding of these stimuli in memory, which gave rise to increased recollection afterwards, or the two effects operated independently of each other (or simultaneously in parallel). To explore this question a series of mediation analyses were conducted separately for the human and the robotic conditions. Considering the findings so far, this analysis was more meaningful in the case of recollection performance, as grasping action increased both recollection and preference, but for completeness, models with the familiarity d’ as dependent variables were also explored. The findings are plotted in Figs. 5 and 6. Analyses with familiarity d’_Independence_ are not presented here, as this measure did not produce any significant effects in the previous analyses reported above, therefore a mediation analysis was not meaningful. However, for completeness, the outcomes of the pre-specified mediation analyses, including all other mediation models presented in the manuscript, are summarised in Table S1. (Supplementary Material).

#### Effect of action on recollection mediated by preference

In the human condition, the direct effect of action type (grasp/static) on recollection performance was significant which was consistent with the result from the ANOVA presented previously, *β* = − 0.16, SE = 0.06, *z* = − 2.52, *p* = 0.012, 95% CI [− 0.29, − 0.03]. Importantly, the indirect effect of action type to recollection, mediated by preference, was not significant, *β* = − 0.0004, SE = 0.007, *z* = − 0.006, 95% CI [− 0.03, 0.02]. This suggests that preference did not mediate the differential effect of action type on recollection (Fig. 5). In the robotic-hand condition, the direct effect of action type on recollection performance was not significant (*β* = 0.04, SE = 0.10, *z* = 0.42, *p* = 0.68, 95% CI [− 0.15, 0.26]), consistent with the diminished effect of action on recollection in this condition (see Fig. 4c). Equally, the indirect effect of action type to recollection, mediated by preference, was not significant, *β* = − 0.0005, SE = 0.004, *z* = − 0.15, 95% CI [− 0.04, 0.02].

#### Action effect on familiarity d’ mediated by preference

In the human condition, the direct effect of action type (grasp/static) on familiarity d’ was significant, which was consistent with the result from the ANOVA presented above, *β* = 1.04, SE = 0.41, *z* = 2.56, *p* = 0.011, 95% CI [0.23, 1.75]. Importantly, the indirect effect of action type on familiarity, mediated by preference, was not significant, *β* = − 0.104, SE = 0.12, *z* = − 0.86, 95% CI [− 0.51, 0.06]. This suggests that preference did not mediate the differential effect of action type on familiarity d’. In the robotic-hand condition, the direct effect of action type on familiarity d’ was not significant (*β* = − 0.08, SE = 0.42, *z* = − 0.19, *p* = 0.85, 95% CI [− 1.03, 0.86]), consistent with the diminished effect of action on familiarity d’ in this condition (see Fig. 3c). Equally, the indirect effect of action type to familiarity, mediated by preference, was not significant, *β* = − 0.02, SE = 0.09, *z* = − 0.19, 95% CI [− 0.27, 0.16].

Overall, the findings from the mediation analyses indicate that the effect of action on subsequent memory was not mediated by preference. In other words, the action at encoding affected preference and memory formation independently, presumably engaging parallel processes.

## Discussion

We sought to investigate variations in mimetic desire, as indicated by preference, and memory formation, associated with goal contagion in human versus robotic interactions. In our experimental tasks, we presented a series of objects undergoing either human or robotic grasping actions or were statically displayed. Our hypothesis posited that the nature of the action (grasping or static) during encoding would influence preference as well as subsequent memory performance. Consistent with our expectations, preference increased for grasped objects compared to static ones, with a more pronounced effect observed in the human-hand condition compared to the robotic-hand condition. Additionally, discernible effects on subsequent memory emerged, revealing that grasped objects led to heightened recollection in the human-hand condition, while no such enhancement occurred in the robotic-hand condition. Conversely, static objects resulted in increased familiarity in the human-hand condition relative to the robotic-hand condition. These findings suggest that the action observation network not only impacts the brain's valuation system but also influences the memory network, thereby affecting the depth of memory encoding. Importantly, the influence of goal contagion on memory was found to be distinct from its effect on preference. Further implications of these findings are discussed in the subsequent sections.

### Mimetic desire: action observation and valuation

The modulation of preference due to goal contagion was supported in the present study. Indeed, grasped objects resulted in increased preference ratings relative to static items with the effect being more pronounced in the human-hand condition than the robotic-hand condition. The proposed mechanism to explain the mimetic desire effect^12,13^, relates to an interaction between the action observation network, when one observes an actor’s current goal, and the brain’s valuation system. Indeed, Lebreton et al.^12^ showed that the desirability of an object was significantly heightened when it appeared as the goal of an individual's action, in comparison to when the object was observed as static or associated with an irrelevant action. This involved activation within the action observation network, while the level of connectivity between the motor system and the valuation system was found to be a significant predictor of the extent of preference evoked towards an object (Lebreton et al. 2012)^12^. These prior and present findings, therefore, underscore the critical role played by the interaction between action observation and the value ascribed to an object in shaping the experience of mimetic desire.

The exact reason action observation affects the subjective value ascribed to an object and, therefore, its preference level may be multi-faceted, but one critical objective may be the optimisation of the decision-making process^35^. Indeed, models of the AON have proposed that the network contributes to determine the goals and intentions of others by minimising prediction errors associated with one’s actions and goals^36,37^. By this account, the interaction between the valuation and action observation systems promotes efficient and flexible decision-making^38^, increasing the desirability of an item and therefore promoting it in preference selection.

This is supported not only by the general directions of the effect on preference in the present study, but it is also further reinforced by the more pronounced effect in the case of human versus robotic agents. A plausible explanation for this is that participants, observing a human hand, which shares physical similarities with their own, could more easily assimilate the actions of the actor within their AON. This could lead them to place higher value on items associated with human–human interactions, in contrast to those linked with human–robot interactions. However, this perspective is somewhat at odds with prior research indicating that the AON can be effectively engaged during observations of robotic movements^27,28^. Nevertheless, these effects may not necessarily translate into preference modulation. It is conceivable that the heightened AON response noted in earlier studies could stem from the rarity of robotic agents in everyday life, thus engaging increased attention due to their scarcity. Similarly, earlier fMRI research revealed that participants more readily attribute intentionality to robotic actions in human–robot interactions when they believe the robot is human-controlled^39^. This underscores the complexity of how interactions with robots influence perceptions of intentionality and desirability, aligning with our findings that interactions between humans more consistently produce a mimetic desire effect.

Another factor that warrants further consideration is the physical characteristics of the robotic agent as more or less anthropomorphic. Prior research indicates a complex relationship between a robot's human-like appearance and its likability, encapsulated in the “uncanny valley” hypothesis e.g.,^40,41^. This theory suggests that as robots become increasingly human-like, they initially gain likability, but beyond a certain threshold, they evoke discomfort or dislike^40,42^). This interplay could significantly impact the ability of artificial agents to elicit mimetic desire effects. In our study, we utilized a distinctly mechanical-looking robotic arm, which might not have sufficiently stimulated the interaction between the AON and the valuation system. It is conceivable that a more anthropomorphic robotic agent could produce a more pronounced effect, even within the robotic paradigm. However, it may be the case that as anthropomorphism increases, the response to the agent could follow a non-linear pattern, as predicted by the “uncanny valley” hypothesis.

### Action observation effects on subsequent memory

The current study provides compelling evidence for a significant interaction between goal contagion and memory quality. Specifically, our findings revealed that objects involved in dynamic grasping actions during the encoding phase, in the human-hand condition were recalled more effectively than static objects. This supports and expands upon limited prior research^24^, which has shown that goal-oriented interactions with an object enhance recall and recognition compared to mere observation of a static item. Conversely, Guérard et al.^42^ observed immediate retention benefits with objects, but these effects did not persist over longer periods.

The present study utilised a recognition memory paradigm, allowing us to distinguish between recollection- and familiarity-based memory. We found that goal contagion significantly influences recollection, which means that associative processing during encoding and the creation of richer, detailed memories allowed retrieval of objects receiving a human action^23,30^. This type of memory engages the binding capabilities of the hippocampus^20,43–45^, activity in which has been associated with increased amount of information recalled at retrieval^46^. An intriguing hypothesis, therefore, is that the AON directly affects the memory network and most prominently the hippocampus when goal-directed actions are committed towards an object.

This idea suggests an expansion of existing models of AON function^36^ to encompass memory changes facilitating adaptable behaviour and future decision-making. In this framework, the AON activates the memory network to encode and store goal-related stimuli, with the hippocampus integrating associative details. This process enhances the vividness and strength of the memory for the stimulus, increasing the likelihood of its future recognition and selection. The implications of such a relationship, particularly in advertising, could be substantial, potentially influencing how objects are perceived and remembered in consumer contexts.

An additional key factor to consider is the extent to which the influence of goal contagion on memory is moderated by mimetic desire. This is crucial as our study revealed a parallel between memory recollection and an increased liking for objects that were handled/grasped. This observation leads to the hypothesis that this enhanced preference could influence the mechanisms of memory encoding, resulting in more vivid memories of these objects. However, our study's mediation analysis challenges this idea. It revealed that goal contagion affected both preference and memory processes, but did so independently of each other. From a neuroanatomical perspective, this suggests that the AON may impact both the brain's valuation system, such as the striatum, and the memory encoding processes in the medial temporal lobe and hippocampus, concurrently. While this hypothesis warrants further investigation through neuroimaging and connectivity studies, our current findings offer substantial behavioural and statistical support for this concept.

The lack of engagement of the same memory encoding mechanism in the robot-hand scenario is noteworthy, as evidenced by a reduced impact on memory recollection for objects that were grasped by the robotic hand. This suggests that interactions with artificial agents do not produce the same enduring memory effects as human–human interactions. The absence of a memory effect for stimuli grasped in the robotic condition suggests that attention to grasped objects does not fully explain memory enhancement for these items. This observation supports the alternative explanation that the activation of the (AON) when viewing human hands contributes to improved memory performance. Additionally, even static objects in the human-hand condition were judged as more familiar than those in the robotic condition. At first, this is unexpected since the static condition was consistent across both scenarios; the objects were presented alone, without interaction from any agent. However, this outcome highlights a significant contextual influence on memory encoding processes when comparing human and robotic agents. In particular, objects associated with human interactions tended to enhance familiarity-based encoding, a phenomenon not observed with robotic agents. This suggests the involvement of a memory orientation mechanism that is more active in the context of human–human interactions, even when the actions are not directly related to the objects. Previous studies have also highlighted that stimuli within an expected context are better remembered on the basis of familiarity^22,47^. This is consistent with the current findings, as a human agent interacting with an object is much more expected than a robotic one. In contrast, robotic actions appear less effective in engaging this memory orientation mechanism, resulting in lower familiarity for static objects and reduced recollection for grasped objects.

Taken together and returning to the discussion regarding the effectiveness of robotic agents to engage the AON in a similar way as human agents, our study highlights a notable difference. We observed that robot-human interactions do not yield the same enduring effects on memory orientation during encoding or on the richness of subsequent memory as seen in human–human interactions. Therefore, models proposing similar mechanisms in human–robot interactions^25^ may not fully encompass the extensive influence of the AON on various cognitive aspects, including mimetic desire and memory. For instance, while functional neuroimaging tasks may show similar AON activations when observing human and robotic agents, this does not necessarily imply equivalent AON engagement. The degree of connectivity with other cognitive networks, like the BNS and the memory network, could differ. This proposal warrants further investigation through the use of connectivity methods in functional neuroimaging studies.

### Future directions and limitations

As the sample characteristics may be an important contributing factor especially in social interactions and the engagement of the AON, future research should attempt to replicate the present findings in different populations, such as younger and older participants. Also, greater representation of male participants would be preferable considering the gender difference in social influence^48^. In future studies, static objects could be associated with non-goal directed actions (e.g., actions directed at the table) to determine if such actions alone can enhance preference and memory for the target object. Our previous work, however, suggests otherwise^13^. In this prior study, neither the presence of a hand nor object movement without an agent (e.g., movement by gravity) enhanced object preference. Finally, as neuroanatomical hypotheses related to the influence of AON on valuation and memory networks directly derive from the present study, functional neuroimaging data will be especially pertinent in future studies.

One limitation of the present study is that we did not analyze the kinematic differences between the actions performed by the robot and the human. Variations in movement dynamics, such as speed, fluidity, and trajectory, could potentially influence the observer's perception and memory of the actions. Future research should consider incorporating kinematic analyses to better understand how these factors contribute to the observed differences in memory and preference. Additionally, examining the impact of action kinematics on goal contagion and mimetic desire could provide deeper insights into the mechanisms underlying human–robot and human–human interactions.

## Conclusion

In conclusion, action observation from human agents has substantial impact on mimetic desire as well as more long-term effects on the quality of memory for objects accompanied by human action. Unlike earlier observations proposing similar engagement of the AON by human and robotic agents, the present findings strongly support that human–human interactions are superior in valuation, memory engagement and the creation of detailed associative memories. Apart from the neuroscientific and behavioural implications, the present findings offer practical insights for marketing strategies and inform the social dynamics of interactions with robotic agents.

### Supplementary Information

Supplementary Table S1.

## Data Availability

Data/material and analyses outputs are available on: https://osf.io/fgwvk/?view_only=c134fdee4c0e4ff4b95a808649c5065e.
