# Supplementary material for: Grasp and remember: the impact of human and robotic actions on object preference and memory
Source: Sci Rep. 2024 Aug 27;14:19851. doi: 10.1038/s41598-024-70692-0 (PMC11349890; doi:10.1038/s41598-024-70692-0)
Supplement: Supplementary file 1 — Supplementary Table S1. [file 41598_2024_70692_MOESM1_ESM.docx]

**Supplementary Material**

**Grasp and Remember: The Impact of Human and Robotic Actions on Object Preference and Memory**

**Table S1.** Mediation analyses parameters for all mediation models for the human and robotic hand conditions separately for the different memory performance measures

| **Condition** | **Mediation analysis** | ***β*** | **SE** | **Z** | ***p*** | **95% CI** |
| --- | --- | --- | --- | --- | --- | --- |
| Human | *action🡪preference🡪familiarity d’* | | | | | |
|  | Direct effect (action🡪familiarity d’) | 1.04 | 0.41 | 2.56 | 0.11 | 0.23, 1.75 |
|  | Indirect effect (mediated by preference) | -0.10 | 0.12 | -0.86 | 0.39 | -0.51, 0.06 |
|  | *action🡪preference🡪familiarity d’_Independence_* | | | | | |
|  | Direct effect (action🡪familiarity d’) | -0.09 | 0.47 | -0.19 | 0.84 | -1.0,  0.87 |
|  | Indirect effect (mediated by preference) | -0.02 | 0.12 | -0.19 | 0.84 | -0.3,  0.26 |
|  | *action🡪preference🡪recollection* | | | | | |
|  | Direct effect (action🡪recollection) | -0.16 | 0.06 | -2.52 | 0.012 | -0.29, 0.03 |
|  | Indirect effect (mediated by preference) | -4.6E+01 | 0.007 | -0.06 | 0.95 | -0.03,  0.02 |
| Robotic | *action🡪preference🡪familiarity d’* | | | | | |
|  | Direct effect (action🡪familiarity d’) | -0.08 | 0.42 | -0.19 | 0.85 | -1.03,  0.86 |
|  | Indirect effect (mediated by preference) | -0.02 | 0.09 | -0.19 | 0.84 | -0.27, 0.16 |
|  | *action🡪preference🡪familiarity d’_Independence_* | | | | | |
|  | Direct effect (action🡪familiarity d’) | -0.09 | 0.47 | -0.19 | 0.84 | -1.04,  0.87 |
|  | Indirect effect (mediated by preference) | -0.02 | 0.12 | -0.19 | 0.84 | -0.30, 0.26 |
|  | *action🡪preference🡪recollection* | | | | | |
|  | Direct effect (action🡪recollection) | 0.04 | 0.10 | 0.42 | 0.68 | -0.15, 0.26 |
|  | Indirect effect (mediated by preference) | -6.3E+01 | 0.004 | -0.15 | 0.88 | -0.04,  0.02 |

*Note:* *β* = parameter estimate, SE = standard error, CI = confidence intervals.
